# Reducing costs for DNA and RNA sequencing by sample pooling using a metagenomic approach

**DOI:** 10.1186/s12864-022-08831-y

**Published:** 2022-08-24

**Authors:** Marc Teufel, Patrick Sobetzko

**Affiliations:** Philipps Universität Marburg, Synthetic Microbiology Center Marburg (SYNMIKRO), Marburg, 35043 Germany

## Abstract

DNA and RNA sequencing are widely used techniques to investigate genomic modifications and gene expression. The costs for sequencing dropped dramatically in the last decade. However, due to material and labor intense steps, the sample preparation costs could not keep up with that pace. About 80% of the total costs occur prior to sequencing during DNA/RNA extraction, enrichment steps and subsequent library preparation. In this study, we investigate the potential of pooling different organisms samples prior to DNA/RNA extraction to significantly reduce costs in preparative steps. Similar to the common procedure of ligated DNA tags to pool (c)DNA samples, sequence diversity of different organisms intrinsically provide unique sequences that allow separation of reads after sequencing. With this approach, sample pooling can occur before DNA/RNA isolation and library preparation. We show that pooled sequencing of three related bacterial organisms is possible without loss of data quality at a cost reduction of approx. 50% in DNA- and RNA-seq approaches. Furthermore, we show that this approach is highly efficient down to the level of a shared genus and is, therefore, widely applicable in sequencing facilities and companies with diverse sample pools.

**Supplementary Information:**

The online version contains supplementary material available at (10.1186/s12864-022-08831-y).

## Introduction

DNA and RNA sequencing is a standard procedure in a wide range of applications. It is applied to identify diseases and novel pathogens as well as to understand the internal regulatory state of a cell or its replication dynamics [1]. In the last decade, RNA and DNA sequencing prices dropped significantly. Further drops in costs are expected to be realised by better sequencing devices with higher read density and read length per chip [1–3]. The drop in price has lead to deep sequencing applications and higher standards which increased total samples and read amounts per study. Consequently, DNA and RNA sequencing still is a major cost factor. The price reduction has reached a point, where the final library sequencing only covers 20% of the total costs of a 10 million read sequencing project, starting from sample preparation after sampling. This implies that efforts for cost reduction have limited effects unless for special approaches requiring extensive read amounts. Considering the 80% costs not covered by library sequencing, efforts could be made for a reduction. However, these costs are linked to chemicals and sample treatment that still requires human labour. Here, further automation could be an option for future cost reduction. However, since samples are not standardised due to their different origin, automation is also limited. In the early stages of DNA and RNA analysis, replicates were pooled on the sample or purified RNA/DNA level to stabilise results at reduced costs [4, 5]. This results in the loss of statistical power due to the lack of independent replicates. Here, we present an approach to pool samples of different organisms and to separate DNA/RNA reads bioinformatically after pooled sample preparation and sequencing. With the pooling of three organisms, total costs for DNA and RNA sequencing could be reduced to 50%. The approach follows the idea of metagenomics [6–8] or in-situ host/pathogen [9–11] studies that have already been shown to generate valuable data from mixed samples. In such approaches, usually the individuals that need to be separated are evolutionary very distant or only a few markers are needed to determine relative numbers of organisms e.g. in the intestines, skin or infections. Modifications to the alignment, quantification, and downstream analysis steps further improve data quality and yield [12]. In this study, the limits of sample pooling for high quality applications in genomics and transcriptomics with respect to error rates and evolutionary distances are investigated.

## Material and methods

### Sampling conditions

Bacteria were grown in rich LB medium at 37 ^∘^C for *E. coli* and *V. natriegens* and 30 ^∘^C for *D. dadantii* in baffled flasks with an air:medium ratio of 4:1. All samples were taken at an OD_600_ of 0.3, pelleted immediately at 7000 rcf for 3 minutes, resuspended in dehydration solution (RNAlater) and kept at 4 ^∘^C over night. For pooling *D. dadantii*, *E. coli* and *V. natriegens* were mixed 1:2:2 in terms of total ODs. DNA and RNA isolation as well as sequencing was performed by a single supplier. The initial sample amounts used in sampling of each organism depended on the required cell pellet amounts matching the supplier criteria. Isolation of bacterial genomic DNA was performed according to Bruhn et al.[13]. For RNA-sequencing, lysis of cells and subsequent isolation of total RNA was carried out using the lysing matrix B/FastPrep *Ⓡ* sample preparation system (MP Biomedicals) and the miRNeasy Mini Kit (Qiagen), respectively. Ribosomal RNA depletion (RNA) and library preparation (RNA/DNA) was conducted by Eurofins Genomics using the Illumina Technology (strand-specific; paired-end; 2x150bp read length).

### Read separation

For read separation, the replicon fasta files of each organism were merged to a single file containing all replicons, in total four, one for *E. coli*, (NZ_CP032679.1) one for *D. dadantii* (NC_014500.1) and two for *V. natriegens* (NZ_CP009977.1, NZ_CP009978.1). Reads of the pooled samples were mapped onto the hybrid fasta using the QuasR library for R. Map settings were set to unique best matches with max 1 mismatch. This means, that only mapped reads were counted where no second match with the same number or less mismatches was found on any of the provided replicons. Coverage of individual replicons was determined and exported using wigExport of the QuasR library. The coverage files of the individual replicons were assigned to the corresponding organism. Single sample experiments were treated equally using genome fasta files of the individual organisms.

### Copy number determination

DNA coverage of the individual replicons were split in consecutive 5000 bp windows and the average coverage was determined within each window. The annotated origin or replication was used to determine the position of the terminus region (ter) on the opposite side of the chromosome. As copy numbers increase exponentially towards the origin of replication, log coverage values were determined to get linearised copy numbers. A linear regression was then performed on the linearised coverage averages of the left and the right replichore separately and regression curves were transformed back to exponential. The abscissa of the intersection of the regression lines of both replichores determined the copy number of origin of replication and terminus. Still representing only DNA dosage in reads, both values were corrected by the terminus value to get a copy number of one for ter and the correct copy number of the origin of replication.

### Pooling error determination

In a first step, 20 organism pairs with equal species, genus, family, order, class and phylum were randomly selected from the NCBI bacteria database. This means that both organisms in a pair were identical e.g. in its family but not on the lower phylogenetic hierarchy (genus). Moreover, each pair was chosen from a random branch of the phylogenetic tree spreading samples all over the bacterial kingdom. For each of the organisms synthetic reads were sampled by moving a sliding window of 150 bp around its replicons with a shift of one. This ensured a full coverage of each locus. For determination of the error introduced by single sample mapping, synthetic reads were mapped to the single organism of the pair to determine the number of uniquely mapped reads. These reads were compared to the total number of synthetic reads provided. To determine the additional error introduced by pooling, the uniquely mapped reads of the single organism were then compared to the uniquely mapped reads obtained by mapping on all replicons of both organisms.

### Tool for read loss analysis

To determine the loss of reads for a given set of species a tool was implemented. The following steps are required for the analysis. 1) create a folder and copy genome fasta and gff files of each organism in the folder. Make sure all fastas end with ‘.fna’ and all gffs end with ‘.gff’. The corresponding fasta and gff files should be identical e.g. ‘E_coli.fna’ and ‘E_coli.gff’. If not, rename the files. 2) download ‘read_loss.jar’ from supplemental data. 3) run ‘read_loss.jar’ with the following command line: ‘java -jar read_loss.jar [path_to_gff_and_fasta_folder] [read_length]’. Gene list files and general read loss file will be created in the gff and fasta folder.

## Results

### Pooling of three organisms

**Fig. 1:**
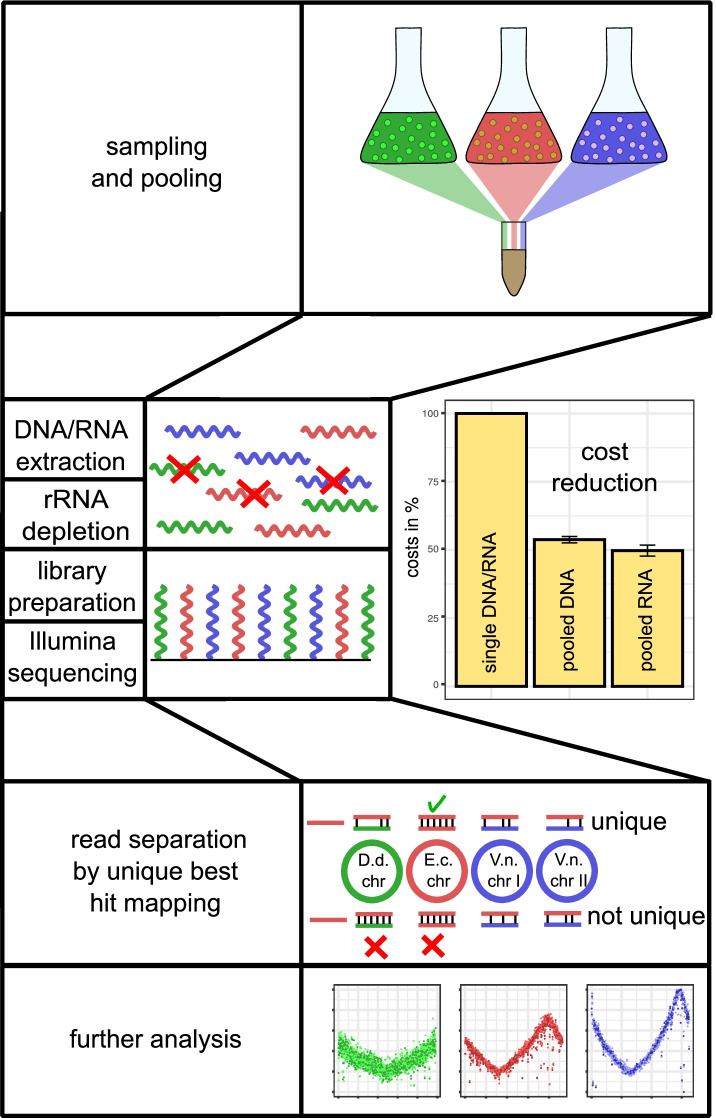
Pooling workflow. By pooling samples of different species, costs can be significantly reduced in DNA/RNA extraction, rRNA depletion and library preparation and sequencing. Costs for single samples are set to 100%. Costs of three companies for pooled samples are relative to single sample costs for DNA-seq and RNA-seq. Error bars indicate standard deviation of costs

The concept of the presented approach is based on the diversity of DNA/RNA between organisms. Hence, it should be possible in theory to assign an organism to each read, as long as both organisms do not share the sequence of that read. Consequently, biological samples can be in principle pooled prior to sample treatment and RNA/DNA isolation and reads can be assigned to the correct organisms after sequencing. The sample preparation prior to sequencing including DNA/RNA extraction, rRNA depletion and library preparation represents a high proportion of total costs (see Table 1, Fig. 1).

**Table 1:** Price distribution of DNA-seq and RNA-seq of three suppliers

Step	Costs % DNA/RNA
DNA/RNA extraction	10-15% / 15-20%
rRNA depletion	0 / 20-30%
library preparation	50-60% / 30-40%
150bp/10M/paired-end reads	20-30% / 30-40%

For pooling of three organisms, sequencing offers of three companies showed a cost reduction of about 50% for the pooled samples compared to single samples with the same type and amount of guaranteed reads and sample preparation(see Fig. 1). For a realistic test, three related organisms within the gamma-proteobacteria (class) were chosen. This clade harbours many frequently sequenced organisms including various pathogens and species relevant for biotechnology. The three organisms comprised the best studied model organism *Escherichia coli*, the fastest growing bacterium *Vibrio natriegens* and the plant pathogen *Dickeya dadantii*. With *E. coli*, an organism was chosen, that is frequently used in biotechnology and basic research and is, thus, sequenced frequently. *V. natriegens* is a promising candidate for future biotechnological applications due to its high growth rate and complex metabolism [14]. Furthermore, it harbours a secondary chromosome providing a chassis for synthetic pathways separated from the main chromosome. *D. dadantii* is a plant pathogen and causative agent of soft rot disease for agriculturally relevant crops including onions, potato, tomato and tobacco [15].

Two replicates of all organisms were grown in rich medium under aerobic conditions at organism specific optimal temperatures. Samples were taken during exponential phase (OD _600_= 0.3) and split for pooled and single analysis. For pooled analysis equal OD_600_ amounts of *E. coli* and *V. natriegens* and half of *D. dadantii* were mixed after sampling. The reduced amount of *D. dadantii* was chosen to investigate quality effects of unbalanced mixing. Single and mixed cell pellet samples were sent for DNA/RNA extraction and 150 bp paired-end Illumina-sequencing by a single supplier. For single samples 5 million reads were ordered. For *D. dadantii* 2.5 million reads were randomly removed to reflect read amounts in the pooled samples. For pooled samples 15 million reads were ordered to reflect single samples read amounts. Sequencing results of the single experiments were then mapped on the respective genome. For pooled experiments, sequencing reads were mapped against all organisms annotated as separate chromosomes of a single hypothetical hybrid organism (see Methods). Only reads were counted that had a unique best match. This means that the read did not align to more than a single chromosomal location with the same number of matching base pairs. The total number of allowed mismatches were set to 1 to keep computation time reasonable. However, allowing for more mismatches (2 and 3) had no negative impact on the results presented in the study and total reads recovered only increase marginally due to low error rate of Illumina sequencing. In a second step, replicons and mapped reads were reassigned to the three initial organisms (see Fig. 1). Total reads of single and pooled samples reflected the guaranteed number of reads and no issues during external preparation were reported. For pooled samples, the relative abundance of organism specific reads reflected the mix ratio 1:2:2 (D. d. : E. c. : V. n.) during pooling (see Fig. S1).

**Fig. 2:**
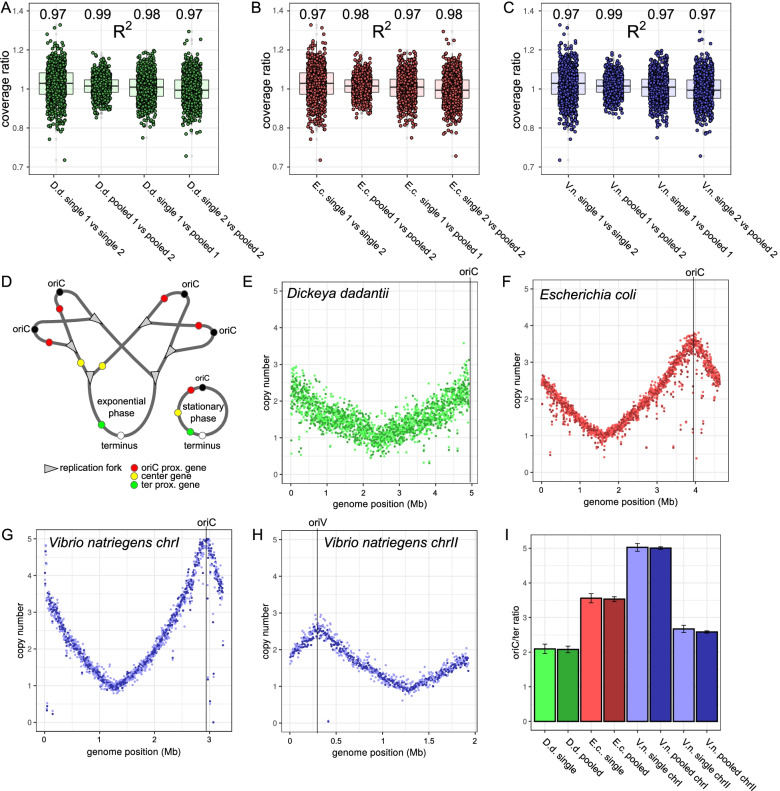
Comparison of DNA-seq data for pooled and non-pooled samples. **A ***Dickeya dadantii* percentage deviation between the local chromosomal coverage (5 kb window) of different sample combinations. R^2^ values of the compared local chromosomal coverage are indicated above individual columns. **B ***Escherichia coli* percentage deviation between the local chromosomal coverage (5 kb window) of different sample combinations. R^2^ values of the compared local chromosomal coverage are indicated above individual columns. **C ***Vibrio natriegens* percentage deviation between the local chromosomal coverage (5 kb window) of different sample combinations. R^2^ values of the compared local chromosomal coverage are indicated above individual columns. **D** Scheme of gene copy numbers caused by overlapping replication rounds. **E** Marker frequency analysis of exponentially growing *Dickeya dadantii* cells. Colors of the data points indicate the two replicates. **F** Marker frequency analysis of exponentially growing *Escherichia coli* cells. Colors of the data points indicate the two replicates of pooled samples. **G** Marker frequency analysis of chromosome I of exponentially growing *Vibrio cholerae* cells. Colors of the data points indicate the two replicates of pooled samples. **H** Marker frequency analysis of chromosome II of exponentially growing *Vibrio cholerae* cells. Colors of the data points indicate the two replicates of pooled samples. **I** Copy number effect between *oriC* and the terminus region during exponential growth derived from pooled and single samples. The ordinate represents the fold change of copies of oriC or oriV relative to the terminus region

To determine the quality of single and pooled samples, DNA coverage distribution along the chromosomes was investigated within 5000 bp windows. A deviation of local coverage between single and pooled samples would indicate problems with read separation and limited applicability for the chosen organisms. For all organisms, sample differences were similar for single vs single compared to single vs pooled indicating no significant loss of data (see Fig. 2 A-C). For the 2-fold reduced set of *D. dadantii* reads, sample deviation did not differ significantly for single vs single and single vs pooled. Hence, 2-fold sample reduction in *D. dadantii* did not affect quality in pooling nor single samples for this analysis. The data was then further analysed to determine the stability of chromosomal copy numbers, a frequently analysed parameter derived from DNA-seq data. DNA replication consumes a large part of the bacterial life cycle. Due to the limited speed of replication, regions close to the origin of replication (oriC) are copied significantly earlier than regions on the opposite side of the chromosome (ter region). Hence, oriC-proximal regions tend to have more copies than oriC-distal regions in a bacterial population (see Fig. 2 D). For many bacteria overlapping replication rounds are possible that increase the discrepancy to 4-8 fold differences between oriC and the terminus region (see Fig. 2 D). This results in a higher amount of oriC-proximal reads compared to oriC-distal reads. The slow growing *D. dadantii* showed the lowest copy number effect followed by *E. coli* and the fastest growing bacterium *V. natriegens* (see Fig. 2 E-H). The lower copy number of chrII compared to chrI of *V. natriegens* is due to a systematically delayed replication initiation of the smaller chrII to achieve synchronous termination [16]. Considering reliability of the data, copy numbers determined from single and pool samples showed consistent values without significant deviation (see Fig. 2 I).

**Fig. 3:**
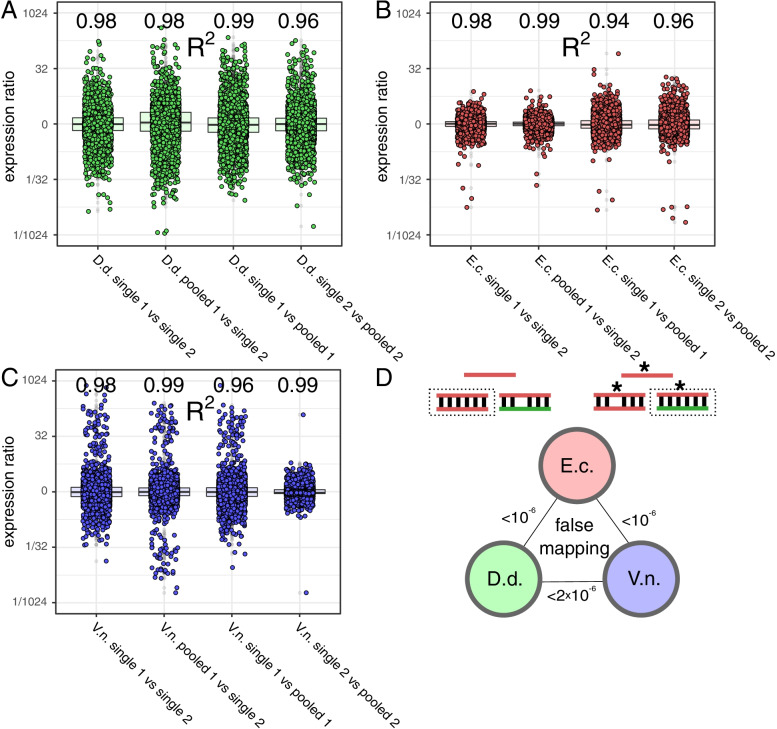
Comparison of RNA-seq data for pooled and non-pooled samples. **A ***Dickeya dadantii* expression ratio between genes of different sample combinations. R^2^ values for gene expression of compared data sets are indicated above individual columns. **B ***Escherichia coli* expression ratio between genes of different sample combinations. R^2^ values for gene expression of compared data sets are indicated above individual columns. **C ***Vibrio natriegens* expression ratio between genes of different sample combinations. R^2^ values for gene expression of compared data sets are indicated above individual columns. **D** Assignment of reads to the wrong organism in pairwise pooling caused by sequence errors in RNA-seq data. 10 ^−6^ indicates a lost read in one million reads. The asterisk indicates the position of the read error. The dotted box indicates the selected match of the read. Colors indicate the origin of the reads and the mapped genome

To verify the results for gene expression data and for later analysis of the impact of sequencing error-rates, RNA-seq was performed using aliquots of single and pooled samples used for DNA-seq. In analogy to the DNA-seq data, RNA isolation and sequencing was performed by the same supplier and mapping was performed with the identical mapping parameters. Gene expression ratios between single and pooled samples show no significant difference or pattern compared to ratios between replicates of single samples(see Fig. 3 A-C). Hence, also RNA-seq can be performed with pooled samples. However, for reduces *D. dadantii* RNA samples, the overall deviation of the ratios increased in all *D.dadantii* samples compared to the other two organisms. Therefore, a good estimation of sample ratios is required to achieve equal amounts of reads per organism if high precision is required. In contrast to the 5000 bp windows in DNA-seq analysis, gene lengths are much smaller (approx. 1000 bp on average). Consequently, more reads are needed to achieve the same statistical robustness of single gene analysis.

### Testing the limits of sample pooling

So far, qualitative analysis revealed that no significant difference or bias could be detected in processed DNA and RNA data. With the confidence of reproducible and high quality data, a quantitative analysis of sample pooling including limits and errors was performed. RNA-seq contains a reverse transcription step for cDNA library preparation that may add more errors to the process than DNA-seq. Therefore, RNA-seq data was used for error analysis. Error-free reads of one organism would never get a better match for another organism, as the read maps perfectly to the originating organism. In worst case, a read maps perfectly to more than one organism, which is addressed later. However, real samples could contain errors and some reads may then map to the wrong organism. Therefore, reads of one organism from the single samples were mapped against the originating organism together with one of the other two organisms. The number of reads that mapped to the wrong organism were determined and compared to the total amount of reads in the sample (see Fig. 3 D). The three pairwise comparisons of two replicates each indicate that such false mappings due to read sequence errors are very rare with a rate of about one in a million and will, therefore, not significantly affect downstream analysis. This low rate is reasonable, since only very few errors occur during RNA-seq [17] and these few errors have to be located at specific locations with the correct base flip within the 150 bp read to trigger a false mapping. With a low probability of a read error and the low probability to occur in the right position and the right base flip, these events are rare. According to the data, for a 10 million reads run, 10 randomly distributed false reads would be expected. Therefore, sequencing errors do not contribute significantly to pooled data quality.

In addition to the false assignment of reads to organisms, reads can be lost due to ambiguous mapping caused by intra-species repeats for single organism samples and homologies between organisms for the pooled samples. To quantify the potential loss of data, simulations were performed. Real data contains artifacts of the library preparation and sequencing process and reads of certain genomic regions are statistically over or underrepresented. Hence, loss of data cannot be reliably quantified. For each of the three organisms, reads were generated using a sliding window of 150bp with a slide of one base pair leading to a single fold coverage of read start positions on each replicon. Consequently, all possible reads of 150bp length are present exactly once in the data set. There are theoretically two different types of read loss. 1) ambiguous mapping within a single organism due to chromosomal repeats. This type of problem occurs in classical single mapping as well as pooled mappings. 2) ambiguous mapping between the pooled organisms due to shared homologies. This may occur preferentially between closely related organisms. In analogy to the DNA-seq data, simulated reads were mapped onto the replicons of the three organisms separately (single) and simultaneously to a hybrid organism containing the replicons of all organisms (pooled). The read counts for single and pooled organisms were detected and compared to the total amount of reads. As all simulated reads have at least one mapping position, the total amount of reads is the reference result for optimal mapping. Pooled samples combine the loss of reads by intra-organism repeats and inter-organism homologies. Therefore, the additional loss of reads due to pooling is the difference between single and pooled sample mapped reads. For the three organisms intra-organism and inter-organism read loss comprised 1.44% and 0.049% for *D. dadantii*, 1.7% and 0.053% for *E.coli* and 1.09% and 0.0045% for *V.natriegens*, respectively. Interestingly, homologies within each organism contributed an order of magnitude more to the loss than the pooling process. As a control, two very closely related organisms, two strains of *E.coli*, *E.coli* MG1655 and *E.coli* DH5 *α* were analysed. In this case, intra-organism and inter-organism read loss of MG1655 comprised 1.7% and 40.7%. For *E.coli* DH5 *α* intra-organism and inter-organism read loss yielded 1.64% and 38.7%. Hence, in the case of very close evolutionary distance, loss of reads due to pooling increases drastically.

**Fig. 4:**
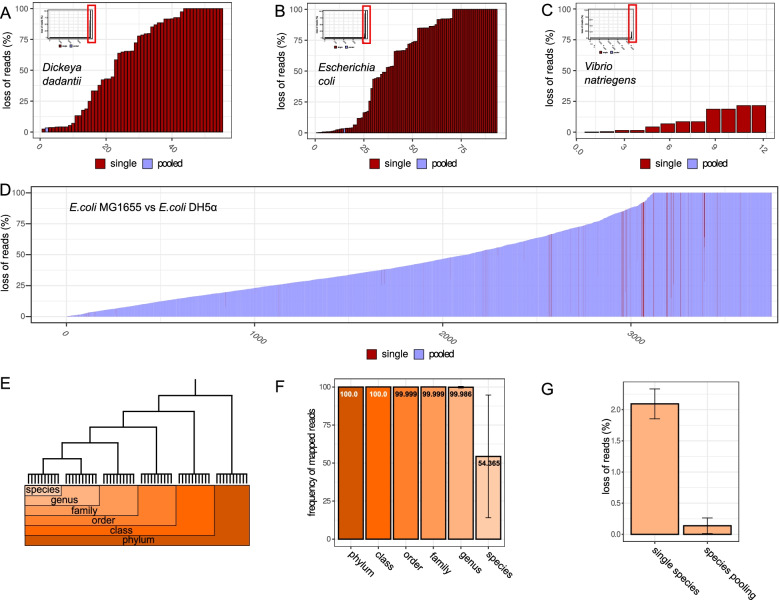
Loss of reads for single and pooled samples. **A** Depicted are read losses for individual genes in *D. dadantii* sorted by the total loss in the pooled sample of *D. dadantii*, *E. coli* and *V. natriegens*. The large plot shows the subset of genes that show a loss of reads. The small plot within the large plot shows the full set of genes and its losses. The red box indicates the region of the large plot within the small plot. The total loss is partitioned in the loss due to repeats within a species (red) and the loss due to pooling with the other two species (blue). Loss of reads per gene in percent for *D. dadantii* pooled with *E. coli* and *V. natriegens*. **B** Loss of reads per gene in percent for *E. coli* pooled with *D. dadantii* and *V. natriegens*. **C** Loss of reads per gene in percent for *V. natriegens* pooled with *E. coli* and *D. dadantii*. **D** Loss of reads per gene in percent for *E. coli* MG1655 pooled with *E. coli DH5 α*. **E** Scheme of comparison of pooled species with different evolutionary distances. **F** Read recovery for pooled samples with species of different evolutionary distances. Error bars indicate standard deviation of 20 random pairs within the NCBI bacteria data base. **G** Loss of reads by classical mapping and additional loss by pooling for 20 unique species pairs within the same genus (genus differs in each pair)

The relatively small loss of reads may still cause problems due to a non-homogeneous distribution on the genome. Consequently, single genes may lose much more of its reads due to large homologies causing false expression values for pooled RNA-seq experiments. Therefore, read mapping and loss was resolved on the gene level (see Fig. 4 A-D). Again, the loss due to pooling was minimal. In the worst case for all genes in all three organisms, 3% of one gene was lost due to homologies between organisms whereas multiple genes were severely affected by intra-organism repeats relevant for pooled and single samples. In the control case, of two *E.coli* strains, many genes were fully lost due to inter-genomic homologies. In this case, pooling is not recommended. The suitability of a specific group of organisms for pooling can be determined with the provided tool (see supplemental material and Methods). It provides lists of read loss for individual genes and the full organisms with respect so single sample sequencing and pooled sequencing. This information can also be used to correct read amounts for individual genes in single and pooled RNA-seq data.

So far, organisms from the Gamma-proteobacteria were tested. Hence, the evolutionary distance is on the phylogenetic level of a class or even order for *E. coli* vs *D. dadantii*. For this distance, no relevant losses of genes were detected. However, for two *E. coli* strains, the pooling approach was not applicable. Hence, there is a minimal evolutionary distance where sample pooling can be applied safely. To systematically test the limits of evolutionary distance for proper read separation, 120 random organism pairs with evolutionary distance of type species (e.g. different strains), genus, family, order, class and phylum were selected (see Fig. 4 E). Analysis was done in analogy to the three previous organisms with the difference that only pairs were pooled. Except for poolings of the same species, recovery was almost complete (see Fig. 4 F). However, homology between strains of the same species varied strongly. Hence, there are cases in which also individual strains of the same species can be pooled. For the closest phylogenetic distance with excellent read recovery (genus), loss of reads due to intra-genomic repeats and loss of reads due to homologies between organisms was determined. Similar to the analysis of the three organisms, the loss present in standard single sample mapping due to intra-chromosomal repeats was more than 10 times higher than the additional loss due to pooling even for more closely related organisms (see Fig. 4 G).

## Discussion

In this study, the limits of sample pooling to reduce sequencing costs, were investigated. For a set of three pooled organisms, costs could be reduced to about 50% of standard single sample sequencing with comparable read yields. Systematic analysis of organism pairs with different evolutionary distance showed potential for successful read separation down to the level of a shared genus. As expected for different strains of a species, pooling turned out to be no reliable option. However, from the level of genus, almost all reads could be correctly assigned to the different organisms. Analysis of DNA and RNA samples of three different organisms indicated no loss in quality of the primary sequencing data and inferred parameters. Consistent with this observation, even at small evolutionary distances (genus), the additional loss of data due to pooling is about 10-fold lower than the loss of data due to repeats in a standard single organism sequencing.

Furthermore, with one in a million false mappings, sequencing errors do not affect data quality of pooled samples significantly. Similar to ambiguous mappings due to inter-organism sequence homologies, mismapping due to sequencing errors relies on shared homologies. Mismapping should, therefore, also pose no threat to pooled sample approaches down to the genus level. In summary, sample pooling yields high quality data and can, hence, be used for common genomics and transcriptomics analysis including genome sequencing, SNP analysis and expression profiling.

The total read amount can have a strong impact on data quality if precision is required. Therefore, samples have to be properly balanced to avoid quality reduction for a underrepresented organism or ordering of additional reads. The analysis of the three organisms has shown that sample ratios are reliable and can be predicted by OD measurements, cell counting or other organism specific sample quantification methods.

In summary, analysis of real data supports reliable read recovery that allows for usual DNA- and RNA-seq applications without relevant loss of data. The robustness of the results suggests that the number of pooled organisms can be increased even further to reduce costs. As the yields of isolated and depleted DNA/RNA, or the DNA/cDNA libraries are no limiting components in the process, costs should consistently drop with the pooling of more organisms.

In this study, bacteria were investigated exclusively. However, the approach can in principle be applied for archea or eukaryots. However, for eukaryots, the average number of reads required is significantly higher than for prokaryots and cost reduction is less effective in these cases. Concerning the timing aspect of samples, samples can be stored for weeks or months due to cheap commercially available RNA stabilisation agents. Samples and even tissue stored in such solutions are easy to mix in defined ratios to pool samples accurately. Hence, samples for pooling do not need to be prepared synchronously but may be collected for weeks before pooling in proper combinations. Furthermore, varying organism composition of sample pools in facilities are only of a concern if one organism exceeds 1/n of the total sample pool. Hence, with 3 organisms, a single species should not cover more than 1/3 of the total samples currently processed by the facility. For a facility with diverse samples, this situation is expected to be rare and can be compensated by pooling only 2 organisms or storage until matching samples arrive. Consequently, the proposed method may be used for labs dealing with different organisms, in sequencing facilities or sequencing companies with an intrinsically high diversity of samples that can potentially be pooled. In general, the larger the pool of samples and the diversity of organisms, the better the presented approach is suited for reducing costs systematically.

Moreover, the proposed sample pooling method is compatible with other cost reduction methods situated at the level of DNA library construction[18, 19]. In this way costs can be further reduced. It is also compatible with different sequencing technologies including SMRT sequencing and nanopore sequencing. These technologies provide long reads that should extend the spectrum of application of pooling, as more repeats are bridged by long reads and the impact of inter-organism homologies are further reduced compared to the 150bp reads used in this study.

## Supplementary Information

**Additional file 1.** Supplemental material.

## Data Availability

DNA and RNA sequencing data for single and pooled experiments is available at the sequence read archive (SRA) by BioProject accession: PRJNA824282.
