# Supplementary material for: Reducing costs for DNA and RNA sequencing by sample pooling using a metagenomic approach
Source: BMC Genomics. 2022 Aug 24;23:613. doi: 10.1186/s12864-022-08831-y (PMC9400246; doi:10.1186/s12864-022-08831-y)
Supplement: Supplementary file 1 — Additional file 1. Supplemental material. [file 12864_2022_8831_MOESM1_ESM.zip › 12864_2022_8831_MOESM1_ESM/species_pooling_final_suppl.pdf]

Reducing Costs for DNA and RNA Sequencing by Sample Pooling using a Metagenomic Approach

Marc Teufel[1] **and Patrick Sobetzko**[1,*]

[1]Philipps Universität Marburg, Synthetic Microbiology Center Marburg (SYNMIKRO), Marburg, 35043, Germany
[*]patrick.sobetzko@synmikro.uni-marburg.de

ABSTRACT

DNA and RNA sequencing are widely used techniques to investigate genomic modifications and gene expression. The costs for sequencing dropped dramatically in the last decade. However, due to material and labor intense steps, the sample preparation costs could not keep up with that pace. About 80% of the total costs occur prior to sequencing during DNA/RNA extraction, enrichment steps and subsequent library preparation. In this study, we investigate the potential of pooling different organisms samples prior to DNA/RNA extraction to significantly reduce costs in preparative steps. Similar to the common procedure of ligated DNA tags to pool (c)DNA samples, sequence diversity of different organisms intrinsically provide unique sequences that allow separation of reads after sequencing. With this approach, sample pooling can occur before DNA/RNA isolation and library preparation. We show that pooled sequencing of three related bacterial organisms is possible without loss of data quality at a cost reduction of approx. 50% in DNA- and RNA-seq approaches. Furthermore, we show that this approach is highly efficient down to the level of a shared genus and is, therefore, widely applicable in sequencing facilities and companies with diverse sample pools.

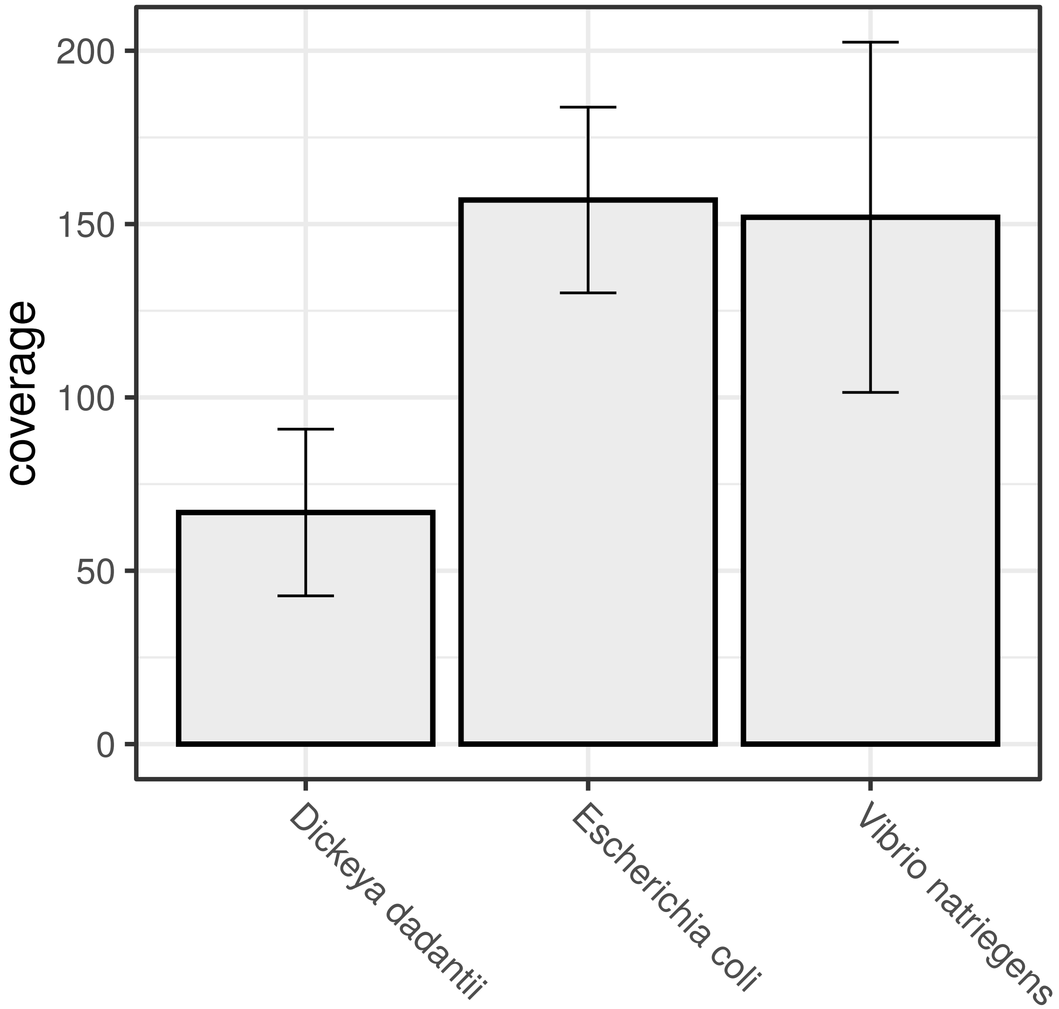

Supplementary Figure 1. Reads extracted from pooling. Coverage of reads after DNA-seq of three species. Only half the amount of *Dickeya dadantii* was added during pooling, resulting in a reduced coverage. Error bars indicate the standard deviation of two biological replicates.
